# Exploring the mechanism of consumer purchase intention in a traditional culture based on the theory of planned behavior

**DOI:** 10.3389/fpsyg.2023.1110191

**Published:** 2023-02-13

**Authors:** Zupan Zong, Xinyu Liu, Huijing Gao

**Affiliations:** ^1^Institute of Cultural Industries, Shenzhen University, Shenzhen, China; ^2^Division of Arts, Shenzhen University, Shenzhen, China

**Keywords:** national tide market, traditional cultural symbols, structural equation modeling, cultural identity, emotional value

## Abstract

There has been a growing interest among scholars and practitioners in cultural empowerment due to the importance of this subject. In this study, we aim to explore the connection between traditional cultural symbols and cultural identity, further estimating how two variables stimulate consumers’ emotional value to generate consumers’ purchase intention. Based on existing traditional cultural literature and the theory of planned behavior (TPB), we first proposed a research framework and then empirically tested the relationship among traditional culture symbols, cultural identity, emotional value, and consumers’ purchase intention. The survey data was tested using structural equation modeling (SEM) and the following conclusions were drawn. First, the cognition of traditional cultural symbols and cultural identity has a direct and significant impact on the emotional value thereby, eliciting consumers’ purchase intention. Second, traditional cultural symbols are directly and indirectly (i.e., through emotional value or cultural identity) positively associated with consumers’ purchase intention, also cultural identity is directly and indirectly (i.e., through emotional value) associated with consumer purchase intention. Finally, emotional values mediate the indirect effect of traditional culture and cultural identity on purchase intention, and cultural identity plays a moderating role between traditional cultural symbols and consumers’ purchase intention. Our findings help to expand the existing literature on consumer purchase intentions by rationally using traditional cultural symbols in the product design and suggesting relevant marketing strategies. The research results can provide valuable inspiration for promoting the sustainable development of the national tidal market and repeating consumers’ purchasing intentions.

## Introduction

In recent years, the term “National Tide” has represented the rise of local trends in China. For example, in the last decade from 2011 to 2021, the search interest for “National Tide” increased by 528%. In 2020, nearly 80% of consumers tend to choose Chinese national brands, and “National Tide” has become an important consumer cultural phenomenon. Among all the existing Chinese national brands, Li Ning is the first well-known sports brand in China, and launched the “China Li Ning” fashion sports series in 2018. The brand of Li Ning targets young consumers, and the collection combined traditional Chinese culture with fashion trends, reversing the existential crisis that has affected the company since 2010. A brand is the carrier of “National Tide,” with traditional culture at the core.

Driven by the trends of modernization and globalization, people are no longer monocultural but rather bicultural or multicultural (Hu et al., 2018). It is established that individual consumption and living habits are influenced by traditional culture and modern culture at the same time. However, traditional culture is the foundation of a country, and self-confidence in a national culture represents an individual’s full affirmation and active practice of the national cultural values (Zlata, 2013; Liu, 2016) and firm confidence in its cultural vitality (Pan et al., 2021). This study comprehensively identified the influence mechanism of traditional culture on purchase intention from the perspective of the theory of planned behavior (TPB).

Shimp and Sharma (1987) introduced the concept of ethnocentrism into consumer behavior research for the first time in the context of economic globalization, and proposed the concept of “consumer ethnocentrism tendency (CET).” They believe that when consumers are faced with the choice of domestic products and foreign products, they will have a preference for domestic products and prejudice against foreign products. This tendency is most pronounced when the group to which they belong is threatened by externally. In addition, Zhou et al. (2010), Wang and Hu (2011) put forward “Chinese National Brand Awareness” for the study of Chinese domestic brands’ purchase intention. These studies mainly focused on the impact of consumer ethnocentrism tendency, economic anxiety, patriotic feelings, and community identity on purchase intentions. However, with the modernization of the economy, globalization, and the networking of cultures, the cultural environment in which individuals live has become more complex and diverse. So, how do cultural symbols affect purchase intention in the consumption behavior of “National Tide” products? How one attitude toward traditional culture affects one’s willingness to purchase “National Tide” products, it is still unknown in the literature.

Our review of the literature revealed various open research gaps. First, prior research on the influence of traditional culture on consumers’ purchase intention is mainly focused on qualitative research designs. Second, there is a lack of measurement scales for traditional cultural identity, and the understanding of the status of traditional cultural identity is only at the empirical and conceptual level. Third, most of the existing research on the influence of traditional culture on consumers’ purchase intention is based on “consumer ethnocentrism tendency.”

Based on the evaluation system of cultural identity, referring to the three stages of cultural identity development proposed by Phinney (1993), this paper divides traditional cultural identity into the cognition of traditional cultural symbols, the confirmation of cultural Identity, and the emotional value of traditional culture. This research adopts a combination of qualitative and quantitative research methods to construct a model of the traditional cultural symbols, cultural identity, emotional value, and consumers’ purchase intention, exploring the mechanism of traditional culture on consumers’ purchase intention. The research results can explain the consumption behavior of national fashion brands and provide enterprises with suggestions and marketing strategies. The rest of this paper is organized as follows: Section “Literature review and hypotheses development” provides a review of the relevant literature. In this section, hypotheses are formulated and the research model is established. Section “Methodology” presents the research methodology, including the questionnaires and data collection involved, and presents the results of the data analysis. Section “Data analysis and hypothesis testing” discusses the findings. Finally, Sections “Discussion and implication” and “Implications” discuss the contributions, theoretical and managerial implications, as well as study limitations and prospects for future research.

## Literature review and hypotheses development

### Traditional culture and cultural identity

Identity includes attribution identity, regional identity, economic identity, cultural identity, political identity, social identity, and national identity (Samuel, 2005). Cultural identity is defined as a “broad range of beliefs and behaviors that one shares with members of one’s community” (Jensen, 2003). Sociologists analyze culture as a system, and discuss the role of culture in creating personal activities, such as the cultural symbols used, the cultural ideas followed, the thinking patterns and behavioral norms (Ann, 1986).

Earlier, Mol divided cultural identity into two dimensions (Powell, 2017). One is to focus on the social dimension. For example, according to Phinney’s definition (1989), cultural identity is an individual’s sense of belonging and psychological commitment to a particular country or nation. Meanwhile, Phinney (1993) divides the development of cultural identity into three stages: unexamined cultural identity, exploration of cultural identity, and achievement of cultural identity. In the unexamined cultural stage, individuals take their own cultural identity for granted, lack awareness of cultural differences, and show little interest in cultural issues. The sense of belonging to one’s group is mainly influenced by factors such as parents, groups, mass media, etc. It is easy to accept and internalize the stereotypes generally held about one’s own group without question. The cultural identity stage is the process of exploring and reflecting on one’s culture, gaining more understanding of one’s own culture, and beginning to understand the meaning of one’s own group identity. This stage is achieved by increasing dialog on cultural issues, reading relevant materials, and participating in cultural activities. At this point, cultural members inject more emotional components into the cultural group, especially when they are antagonistic to other groups. The cultural identity achievement stage is the clear and confident acceptance of the culture, and the cultural identity is internalized into self-awareness. At this stage, people can properly deal with stereotypes and discrimination, enhance cultural confidence, and a positive psychological adaptation (Myron and Jolene, 2010). The other is to focus on the personal dimension of cultural identity, emphasizing “cultural identity.” Erikson (1968) believes that cultural identity refers to the social psychological process by which individuals internalize their own culture and cultural groups and generate a sense of belonging to acquire, maintain and innovate their own culture.

Whether from the social dimension or the individual dimension, cultural identity is not innate and is created in the process of human socialization. Phinney (1991), after reviewing the research on ethnic identity, pointed out that strong mainstream identity is only related to high self-esteem when there is a positive adaptation to mainstream culture. Hirai (2016) pointed out that traditional culture in a macro sense indicates all human activities such as religion, philosophy, moral standards, laws, politics, economics, society, history, literature, and art that have been preserved, learned, and transmitted in each community or group over a long period.

Culture includes traditional culture, but it is not completely consistent. However, there are few related studies on traditional cultural identity. Concepts like traditional cultural identity include “ethnocentrism” (Sumner, 2016), “ethnic consciousness,” and “national community identity” (Zhou et al., 2010), etc. For the evaluation of cultural identity, many studies have designed and developed corresponding measurement tools based on the theory of group and social identity. Phinney (1992) developed MEIM (Multigroup Ethnic Identity Measure), which includes three parts: Affirmation and Belonging, Ethnic Identity Achievement, and Ethnic Behaviors. In the study of Phinney and Ong in 2007, MEIM-R (Multigroup Ethnic Identity Measure-Revised) scale was refined, and only six items were used to measure cultural identity from two dimensions: Exploration and Commitment. In addition, Wang and Hu (2011) divided cultural identity into three dimensions: cultural symbol identity, cultural identity, and cultural value identity. Cultural symbol identity measures the attitude tendency of individuals toward ideographic symbols such as material forms, language, and life matters in different cultural backgrounds; cultural identity is to measure people’s attitude, evaluation, belonging tendency, and emotional attachment to different cultural groups; and cultural value identity examines the degree of individual acceptance of social norms and cultural value of specific cultural groups in terms of self-awareness (Wang and Hu, 2011). Based on the evaluation system of cultural identity, referring to the three stages of cultural identity development proposed by Phinney (1993), this paper divides traditional cultural identity into the cognition of traditional cultural symbols, the confirmation of cultural Identity, and the emotional value of traditional culture.

### Traditional cultural symbols

Cultural symbols include both linguistic symbols and non-linguistic symbols (Rosendaal and Reitsma, 2017). It is the carrier of culture and plays an important role in cultural creation, cultural significance transmission, and inheritance. Culture-based research is characteristic of modern semiotics (Michael and Mark, 1996) and other fields of study such as folklore analysis, anthropology, narratology, disclosure analysis, and art semiotics (Greimas, 2002). Symbols have two main functions: covering “Signification” (Peirce) and “Expression” (Saussure) (Atkin, 2022). As far as cultural symbols are concerned, they are the core meaning system for cultural inner groups to achieve cultural identity, and also the representation system for other cultural groups to identify and communicate with a cultural system (Dwight, 2014; Gong et al., 2020a).

Traditional cultural symbols are the carrier of traditional culture, which can intuitively express national characteristics and national styles, and are the external manifestations of various ideological cultures and ideologies in national history. From the cultural level, traditional cultural elements can be divided into three levels: first, the external “tangible” level representing the physical elements of cultural product design, such as color, outline, texture, heraldic decoration, etc.; second, the medium level include “behavioral” elements with operational characteristics such as functionality and craftsmanship; finally, third inner level represents the “intangible,” which includes intangible psycho-cultural elements such as those with special meaning, a sense of story, and a sense of emotion (Benny and Hazel, 2003).

Chinese traditional cultural symbols are based on thousands of years of historical and cultural ideas and integrate the cultural characteristics of various ethnic groups and regions. Traditional cultural symbols can be divided into explicit symbols and invisible symbols. The explicit symbols include Chinese characters, Chinese calligraphy, the Forbidden City, the Great Wall, Suzhou Gardens, Terracotta Warriors and Horses, Silk, Porcelain, Peking Opera, Shaolin Temple, Kung Fu, and so on. Invisible symbols such as Yin-Yang, Taoism, Confucianism, Zen Buddhism, and so on, are the spiritual connotation implied in various tangible symbols. For example, in calligraphy art, from pen and ink color to structure composition, symbols embody the principle of balance between Yin and Yang. The shade of ink color is a kind of yin and yang: indifference is yang while thick ink tastes yin, and dry ink is yang while moist ink is yin. In terms of composition, it is not only the arrangement of Chinese characters but also the blank space between words and lines that make the whole work balance yin and yang and have a sense of rhythm. No matter what traditional cultural symbols are involved, they all reflect the Chinese people’s outlook on life, values, ethics, customs, cultural education, and the wisdom and philosophy of the Chinese nation. It can be said that traditional culture subtly affects the life of modern people, providing a steady stream of elements and inspiration for product design. In particular, future design needs to return to the perspective of humanities and esthetics, and technology cannot be used as a simple auxiliary tool to guide design (Shi et al., 2021).

According to different types of traditional cultural symbols, the application of product design can usually be divided into two segmentations: one is the representational transformation of symbols, while the other is the abstract transformation of symbols (Gong et al., 2020b; Fang et al., 2021). Representational transformation refers to the direct extraction and transformation of tangible traditional cultural symbols, and their application to product design; for example, cultural and creative products in museums directly apply traditional cultural elements such as images, colors, and lines to daily necessities, showing Chinese esthetics in the overall style. Abstract transformation refers to the transformation of intangible traditional cultural symbols and the application of abstract ideology in product design concepts. The design concept of “tracking,” “realizing” and “walking” clothing and footwear series launched by China Li Ning originated from the wisdom and thinking of traditional Chinese culture. The application of traditional cultural symbols in product design can stimulate patriotic pride in consumers’ hearts and satisfy cultural needs and a sense of belonging. In the field of consumption, the application of cultural symbols can easily stimulate consumers’ cultural identity, which in turn triggers purchase intentions.

### The influence of traditional culture in purchase intention

Purchase intention is rooted in consumer psychology, and reflects the subjective probability that consumers are willing to take a specific purchase behavior (Li et al., 2021; Zhou et al., 2023). Mullet and Karson (1985) believed that consumers’ attitudes toward a certain product or brand, combined with the effect of external factors, constitute consumers’ purchase intention, which can be regarded as the subjective tendency of consumers to choose a specific product. It has also been shown to be an important predictor of consumer behavior (Blackwell et al., 2018).

The Theory of Planned Behavior (TPB; Ajzen, 1991) is considered the most famous theory of attitude-behavior relationship in social psychology. It has been widely used in many behavioral fields and has been proven to significantly improve the predictive and explanatory power of behavioral research. TPB purports that the combination of three core components including attitude, subjective norms, and perceived behavioral control shapes an individual’s behavioral intentions. And put forward the basic hypothesis of the behavior intention model, that is, the more positive an individual’s attitude toward a certain behavior, the stronger his behavior intention; and the more positive the subjective norm of a certain behavior is, the stronger the individual’s behavioral intentions will be stronger. When the attitude and subjective norms are more positive and the perceived behavior control is stronger, the individual’s behavioral intention will also be stronger. The TPB has indicated the change in consumers’ attitudes is the main inducement of the change in consumers’ behavior.

The prediction of consumer behavior is directly related to the sales of products and the decision-making of the company (Jeong and Jin, 2020). Hence, planning behavior is an important research issue in consumption behavior, as well as a major concern of marketing researchers. In addition to consumer attitudes, consumer identity is also closely related to consumer behavior (Mullet and Karson, 1985). As the anthropologist Friedman (2012) pointed out the consumption in the world system is always the consumption of identity. The sociology of consumption holds that identity includes not only the action side, which is related to the paradigm of “consumption behavior,” but also the culture and symbol side, which is related to the paradigm of “cultural consumption” (Rössel et al., 2017). Individual identity and consumption behavior are two aspects of the same process. Personal consumption behavior is not only a reflection of disposable money and resources but also a reflection of personal identification of certain symbols or values. Therefore, consumption activity is a special and important identity behavior. Cultural identity and belonging are affirmed through lifestyles, which are in turn expressed through product conversations and manifestations of related consumer behaviors (Grant, 1986; Søren et al., 2005; Cao et al., 2018; Bodhi et al., 2022). This social interpretation of products is consistent with the theory of planned behavior (Ajzen, 1985).

In consumption behavior, the three original constructs of individual attitude, subjective norm, and perceived behavioral control in TPB are not sufficient to explain the consumers’ purchasing behaviors. Simonson (1992) further expanded the original construction of TPB to include cultural identity, moral norms, emotional values, etc. As mentioned above, the application of traditional cultural symbols in product design not only endows products with unique cultural quality but also increases consumers’ emotional resonance and cultural identity. For example, Shimp and Sharma (1987) pointed out that when consumers are faced with the choice of domestic and foreign products, they will have a preference for domestic brands and prejudice against foreign products, and this tendency is most prominent when their group is threatened externally.

Based on CET (Consumer Ethnocentrism Tendency), Zhou et al. (2010) point out that the consciousness of domestic products is formed under the influence of three factors: national economic hardship consciousness, national patriotic emotion, and national community identity. From the perspective of the national brand community, “Chinese National-brand Consciousness” can be regarded as a phenomenon where the members of a national community have a sense of responsibility for the brands because of their identity as a community, thus generating a supportive awareness of national brands. Nationalism, namely community identity, and patriotic feelings have a positive impact on the consumers’ purchase intention. Grounded on the above findings, this study learns from Shimp and Sharma’s “Consumer Ethnocentrism Tendency” and “Chinese National-brand Consciousness” model, and proposes the following hypotheses from the perspective of traditional cultural identity:

*H1*: The degree of cognition of traditional cultural symbols has a positive impact on cultural identity.

*H2*: The degree of cognition of traditional cultural symbols has a positive impact on the emotional value of traditional culture.

*H3*: The stronger consumers' cognition of traditional cultural symbols, the. stronger consumers' purchase intention of national tide brands.

*H4*: The degree of cultural identity has a positive impact on the emotional value of traditional culture.

*H5*: The stronger the cultural identity of consumers, the stronger the purchase intention of national tide brands.

*H6*: The stronger the emotional value of the traditional culture of consumers, the stronger the purchase intention of national tide brands.

*H7*: Consumers' emotional value identification of traditional culture has a mediating effect on the purchase intention of national tide brands.

*H8*: Consumers' cultural identification of traditional culture has a mediating effect on the purchase intention of national tide brands.

**Figure 1:**
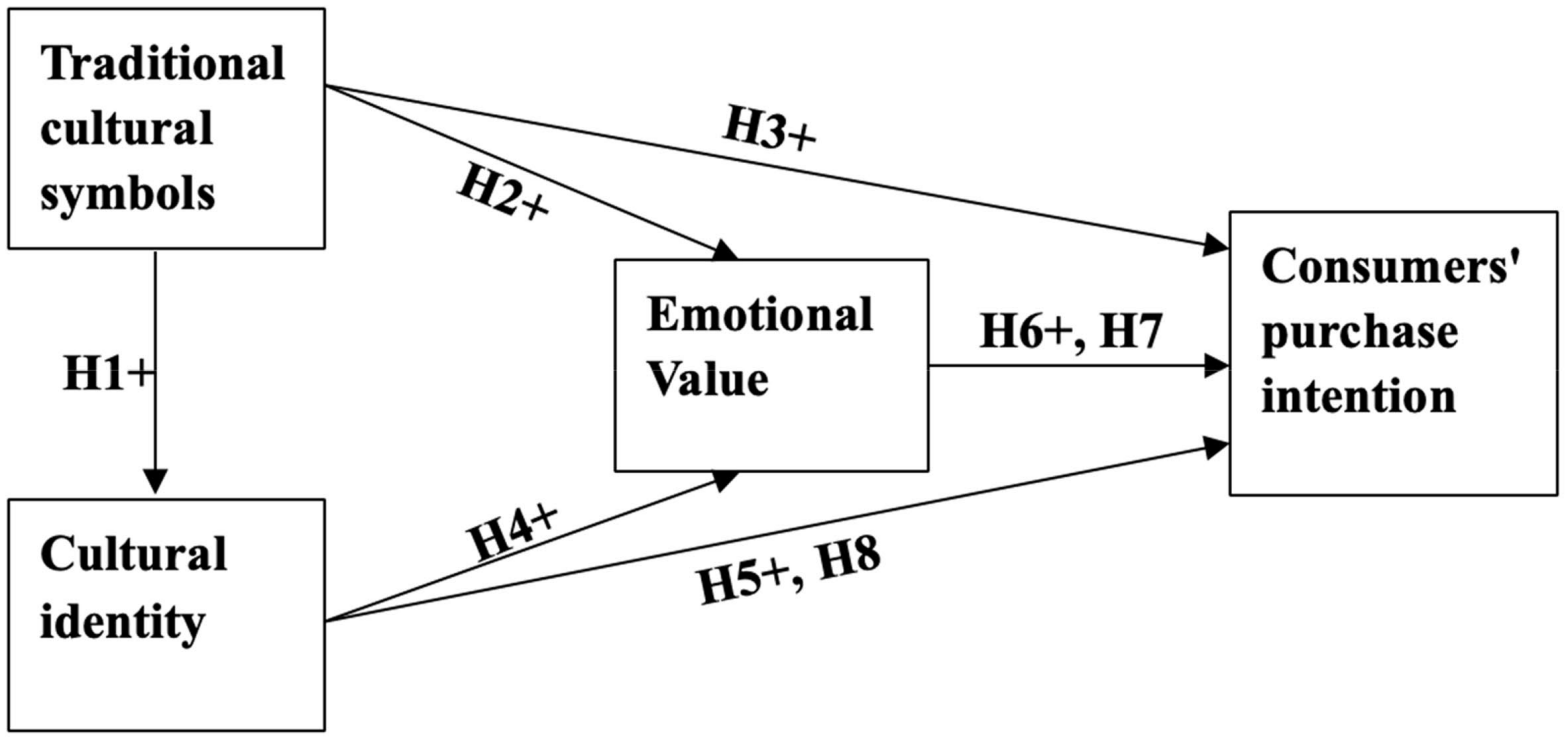
Conceptual model of traditional culture on purchase intention.

Based on the above assumptions, combined with the cultural identity theory, and related research on TPB behavioral intentions, we can construct a conceptual model, as shown in Figure 1. This model explains the internal relationship between consumers’ traditional cultural identity and consumers’ behavioral intentions. Intention to purchase, with the consumers’ emotional value as a mediator.

## Methodology

### Procedures and sample

The brand of Li Ning was selected for the study because it was the first Chinese sports brand and debuted its “National Tide” line “China Li Ning” in 2018. Targeting young consumer groups, the collection combines Chinese traditional culture and elements with fashion trends and reversed the survival crisis affecting the company since 2010. By upgrading its product and brands, Li Ning achieved a revenue of 22,572 billion yuan in 2021 under the COVID-19 epidemic, a year-on-year increase in gross profit of ~68.7%. The success of Li Ning is a typical example of the influence of traditional culture on consumers’ purchase intention. A questionnaire targeting Li-Ning’s consumer behavior is highly representative.

Before formally distributing the questionnaire, 50 students were first selected in the university for a pilot study (e.g., Luqman and Zhang, 2022; Luqman et al., 2022), and the test items whose analysis resulted in <0.5 or higher than 0.5 at the same time were deleted, while the test items that were not expressed clearly and accurately were repeatedly adjusted, and finally, 25 test items were left as the official scale. After the pre-test stage, questionnaires were widely distributed on online communication platforms such as WeChat and QQ. Four hundred and thirty-one questionnaires were collected within 3 days. After screening out some questionnaires with short response times, similar test items but large differences in answers, and multiple repetitions of the same response. Four hundred and five valid questionnaires were finally collected, with an efficiency rate of 93.97%, and the sample data statistics are shown in Table 1.

**Table 1:** Subject’s demographic and usage behavior.

Measure	Item	Frequency	%
Gender	Male	121	29.88
	Female	284	70.12
Age	10–19	22	5.43
	20–29	310	76.54
	30–39	50	12.35
	40–49	13	3.21
	50–59	10	2.47
Occupation	Government staff/Government cadres/civil servant	11	2.72
	Corporate managers (including junior and middle and senior managers)	28	6.91
	Corporate staff	68	16.79
	Professionals (doctor/lawyer/journalist/teacher, etc.)	56	13.83
	Self-employed	7	1.73
	Freelance/Temporary Work	22	5.43
	Current students	204	50.37
	Other (housewives, retired, unemployed, etc.)	9	2.22
Education	High School/Vocational High School	6	1.48
	Junior college	21	5.19
	University	184	45.43
	Master	164	40.49
	PhD	30	7.41
Annual income	Below ¥50,000	212	52.35
	¥50,001–¥100,000	70	17.28
	¥100,001–¥200,000	69	17.04
	¥200,001–¥300,000	26	6.42
	Above ¥300,000	28	6.91
Marital status	Unmarried	341	84.20
	Married	59	14.57
	Divorced	5	1.23

Valid *n* = 405.

Descriptive statistical analysis showed that among all respondents, most of the respondents were female (284), accounting for 70.12% of the total respondents, while the number of males was 121, accounting for 29.88% of the total respondents. The overall age of the respondents was relatively young, with 81.97% of the respondents being under 30 years old. Among the occupations, the majority were students, with 204 respondents (50.37%). Most of the respondents had a bachelor’s degree or above (378, accounting for 93.33%), among which 184 (45.43%) had a bachelor’s degree; 164 (40.49%) had a master’s degree, and 30 people (7.41%) with doctor’s degree. In terms of personal marital status and income, the respondents were mostly unmarried, with 341 people (84.20%), and the income level was mostly below 50,000, with 212 people (52.35%).

### Scale design

This study explored the customers’ traditional cultural identity and purchase intention of “National Tide” brands. A literature scale was designed for this investigation based on the MEIM scale developed by Phinney (1992) in his study (see Table 2). The first part is the measurement of consumers’ awareness of traditional cultural symbols. The second part measured the consumers’ emotional attitudes toward traditional culture such as ‘belonging’ and pride that was based on the scale of the research of Wang (2011) and Zhou et al. (2010). The third part is the measurement of consumers’ purchase intention. Extending previous studies, a questionnaire was designed to grasp the characteristics of the respondents (Luqman et al., 2020b). All the items were measured using a 5-point Likert scale (from “strongly disagree = 1” to “strongly agree = 5”), and the respondents answered according to their actual experiences.

**Table 2:** Measurement items.

Dimension	No.	Description
Traditional cultural symbols	TCS1	I have knowledge of traditional cultural symbols (elements)
	TCS2	Traditional cultural symbols (elements) are attractive to me (including Chinese characters, Confucius, calligraphy, the Great Wall, Chinese medicine, the Forbidden City, the Terracotta Warriors, etc.)
	TCS3	I know Chinese history culture very well (dynastic changes, historical figures, etc.)
	TCS4	I am very familiar with Chinese philosophical thought (Confucianism, Mohism, Taoism, Legalism, etc.)
	TCS5	I know Chinese language culture very well (Chinese, minority languages, poems and songs, etc.)
	TCS6	I know Chinese art culture very well (calligraphy, painting, music, opera, arts and crafts, etc.)
	TCS7	I know Chinese folklore very well (festivals, customs, weddings and funerals, etc.)
Cultural identity	CI1	I spend a lot of time learning to understand traditional culture (history, philosophy, language, art, folklore, etc.)
	CI2	I did not spend much time to understand my cultural background (physical culture such as food, architecture, etc., and spiritual culture such as language, customs, etc., which influence physical and mental development and personality formation)
	CI3	I am well aware of the influence of traditional culture on my life (clothing, food, housing, transportation, etc.)
	CI4	I spend a lot of time sharing and discussing traditional culture with others
	CI5	I like to spend time learning about other cultures (such as Japanese and Korean, European and American cultures, etc.)
Emotional value	EV1	I identify more with traditional culture than with Western culture
	EV2	I have a strong sense of pride in traditional culture
	EV3	I have a strong sense of belonging to traditional culture
	EV4	I do not want to be friends with people from other cultures
	EV5	I have a positive attitude toward the development of traditional culture
Consumers’ purchase intention	CPI1	I am interested in Li Ning products that contain traditional cultural symbols
	CPI2	I am against the combination of traditional culture and fashionable and popular elements in Li Ning products
	CPI3	I am interested in Li Ning products that combine traditional culture and fashionable and popular elements
	CPI4	I have a desire to buy Li Ning products that combine traditional culture with fashionable and popular elements
	CPI5	I think the combination of traditional culture and fashionable elements of Li Ning products have cultural deposits
	CPI6	I think the design of Li Ning products that combine traditional culture and fashionable elements is novel and attractive
	CPI7	I think the combination of traditional culture and fashionable elements of Li Ning products in line with personal esthetics
	CPI8	I think the combination of traditional culture and fashionable elements of Li Ning products in line with the current fashion trends

## Data analysis and hypothesis testing

### Theoretical model and empirical analysis

In this study, SPSS 27.0.1 and AMOS 26 were used to test the correlation of the scales. SPSS 27.0.1 was used to illustrate the population characteristics of the sample and its reliability (Cronbach’s alpha) through descriptive analysis, and AMOS 26 was used to verify the validity of each factor. With gender, age, education level, occupation, annual income level, marriage, and place of residence as control variables, the proposed hypothesis was tested using a structural equation model (SEM) to avoid possible multicollinearity among independent variables (e.g., Luqman et al., 2020a, 2021).

### Reliability and validity test

The data analysis of Table 3 shows that the questionnaire has good reliability. The Cronbach’s alpha values of each scale are above the critical value of 0.07, and the total reliability of the questionnaire is 0.908. Meanwhile, the convergence of the scale was evaluated using the variable correlation coefficient analysis, and the results showed that the CITC values of the analyzed items were all above 0.7, indicating a good correlation between the analyzed items, as well as good reliability. The standardized factor loadings for all measured elements ranged from 0.755 to 0.854, higher than 0.6; therefore, all elements were statistically significant. Table 3 also shows the average variables extracted (AVE) values ranged between 0.608 and 0.651; indicating good validity (Fornell and Larcker, 1981). The square root of the AVE of the latent variables was generally >0.5 (Table 4). At this point, the next step of the analysis can be performed (e.g., Masood et al., 2020, 2022).

**Table 3:** Factor loading, Cronbach’s alpha, CR, and AVE.

Dimension	No.	Factor loading	CITC	Cronbach’s alpha	AVE	CR
Traditional cultural symbols	TCS1	0.792	0.749	0.916	0.608	0.916
	TCS2	0.784	0.74			
	TCS3	0.744	0.711			
	TCS4	0.788	0.749			
	TCS5	0.788	0.752			
	TCS6	0.782	0.743			
	TCS7	0.78	0.742			
Cultural identity	CI1	0.793	0.746	0.903	0.651	0.903
	CI2	0.777	0.737			
	CI3	0.799	0.749			
	CI4	0.811	0.76			
	CI5	0.854	0.796			
Emotional value	EV1	0.776	0.722	0.887	0.611	0.887
	EV2	0.777	0.723			
	EV3	0.776	0.721			
	EV4	0.785	0.734			
	EV5	0.792	0.729			
Consumers’ purchase intention	CPI1	0.775	0.741	0.926	0.609	0.926
	CPI2	0.79	0.758			
	CPI3	0.794	0.759			
	CPI4	0.755	0.722			
	CPI5	0.779	0.748			
	CPI6	0.759	0.722			
	CPI7	0.788	0.756			
	CPI8	0.8	0.766			

AVE, average variance extracted; CR, composite reliability.

**Table 4:** Discriminant validity.

	Traditional cultural symbols	Cultural identity	Emotional value	Purchase intention
Traditional cultural symbols	**0.780**			
Cultural identity	0.456	**0.807**		
Emotional value	0.509	0.463	**0.781**	
Purchase intention	0.517	0.598	0.532	**0.780**

The data on the diagonal is the square root of AVE.

### Hypothesis testing

**Figure 2:**
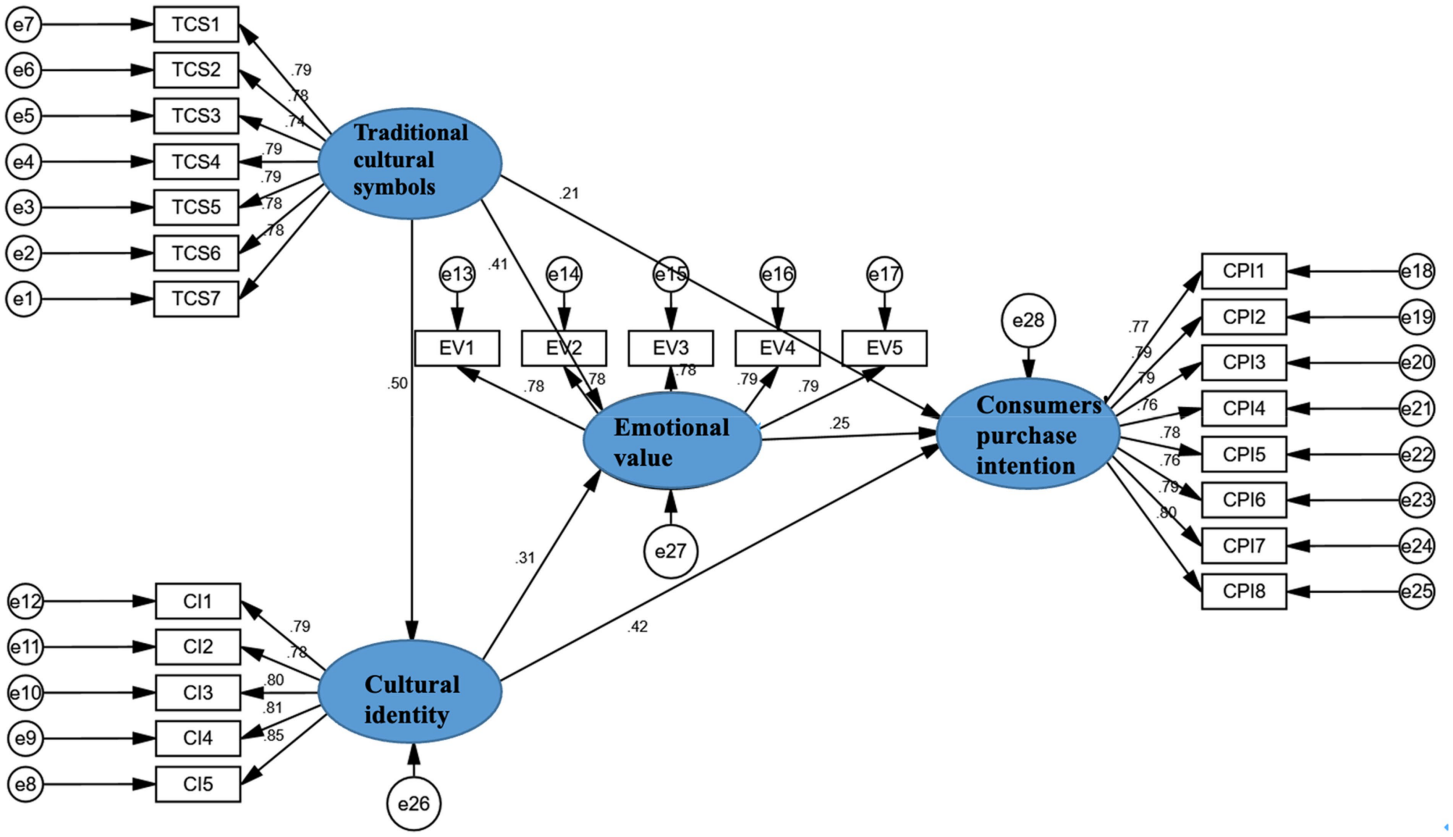
The original edition of structural equation modeling.

Before testing hypotheses, the model fit was tested by using AMOS 26 (Figure 2), and the detailed results are shown in Table 5. The *χ^2^*/df value is between 2 and 3, indicating the validity of the model. The root means square error of approximation (RMSEA) is equal to 0.018; <0.05, the modeling is considered to fit well. IFI (Incremental Fit Index) is 0.954, TLI (Tucker-Lewis Index), CFI (Comparative Fit Index), and GFI (Goodness of a Fit Index) is around 0.9. The results are within the acceptable range of fit indicators but not to the absolute fit, because of slightly lower values compared to good model fit (Michael and Robert, 1992; Jöreskog and Sörbom, 2002; Hair et al., 2009).

**Table 5:** Model fit indices.

Factors	*χ*^2^/df	RMSEA	NFI	IFI	TLI	CFI	GFI
Value	1.136	0.018	0.848	0.954	0.994	0.994	0.945

Table 6 presents the result of testing the proposed hypothesis using SEM. The finding of this survey suggested a positive relationship among traditional culture symbols, cultural identity, emotional value, and consumers’ purchase intention. The estimate of all hypotheses ranged from 0.198 to 0.578, higher than 0, and the critical ratio (CR) is between 3.937 and 9.205, higher than 2. Meanwhile, the *p*-value of all hypotheses is <0.001, indicating that traditional cultural symbols have a direct impact on cultural identity, emotional value, and consumers’ purchase intention. Cultural identity has a direct effect on emotional value, and consumers’ purchase intention. Emotional value has a direct impact on consumers’ purchase intention. Hypotheses H1, H2, H3, H4, H5, and H6 were supported for this study.

**Table 6:** The regression weights of casual paths.

Hypotheses	Estimates	SE	C.R.	Value of *p*	Results
H1	0.578	0.063	9.205	***	Supported
H2	0.4	0.057	6.984	***	Supported
H3	0.198	0.05	3.937	***	Supported
H4	0.265	0.048	5.556	***	Supported
H5	0.342	0.044	7.736	***	Supported
H6	0.24	0.054	4.477	***	Supported

### Mediating effects

To further explore the influence of emotional values on consumers’ purchase intentions, this study used the bootstrap test proposed by Preacher and Hayes (2008), with 2000 sampling times, to verify the existence of the mediation effect, which can be considered significant if the 95% confidence interval does not contain 0 (Zhao et al., 2010). This study used traditional cultural symbols and cultural identity as the independent variable, used the consumers’ purchase intention as the dependent variable, and used cultural identity as the mediator. The results are shown in Table 7. When the emotional value is the mediator variable, traditional cultural symbols are the independent variable, the direct effect is significant (95% confidence interval: 0.102, 0.313), and the mediating effect is significant (95% confidence interval: 0.05, 0.164) with a percentage of mediating effect of 18.18%. It indicates that emotional value plays a partly intermediate role between traditional cultural symbols and consumers’ purchase intention. When the emotional value is the mediator variable, cultural identity is the independent variable, the result indicating that the direct effect of cultural identity on consumers’ purchase intention is significant (95% confidence interval: 0.23, 0.473), and the mediation effect is also significant (95% confidence interval: 0.028, 0.115). This indicates that emotional value plays a partly intermediate role between cultural identity and consumers’ purchase intention. H7 is statistically supported. When cultural identity is the mediator variable, the traditional cultural symbols are the independent variable, the direct effect is significant (95% confidence interval: 0.102, 0.313), and the mediating effect is significant (95% confidence interval: 0.124, 0.301) with a percentage of mediating effect of 38%. H8 is statistically supported. This suggested that cultural identity also plays a partly intermediate role between traditional cultural symbols and consumers’ purchase intention. With the support of Preacher and Kelley (2011), we can suggest that in this study, emotional value plays a relatively small mediating role between cultural identity and consumers’ purchase intention. While the mediating effect of cultural identity on traditional cultural symbols and consumers’ purchase intention is relatively great.

**Table 7:** Test of the potential mediating effects.

Effect category	Effect value	95% confidence interval		Percentage	Conclusion
		Boot CI Lower Bounds	Boot CI Upper Bounds		
Traditional cultural symbols → emotional value → consumers’ purchase intention					
Direct effects	0.198	0.102	0.313	18.18%	Partial mediate
Indirect effects	0.096	0.05	0.164		
Total effects	0.528	0.422	0.649		
Cultural identity → emotional value → consumers’ purchase intention					
Direct effects	0.342	0.23	0.473	16%	Partial mediate
Indirect effects	0.064	0.028	0.115		
Total effects	0.405	0.288	0.533		
Traditional cultural symbols → cultural identity → consumers’ purchase intention					
Direct effects	0.198	0.102	0.313	38%	Partial mediate
Indirect effects	0.198	0.124	0.301		
Total effects	0.528	0.422	0.649		

**Figure 3:**
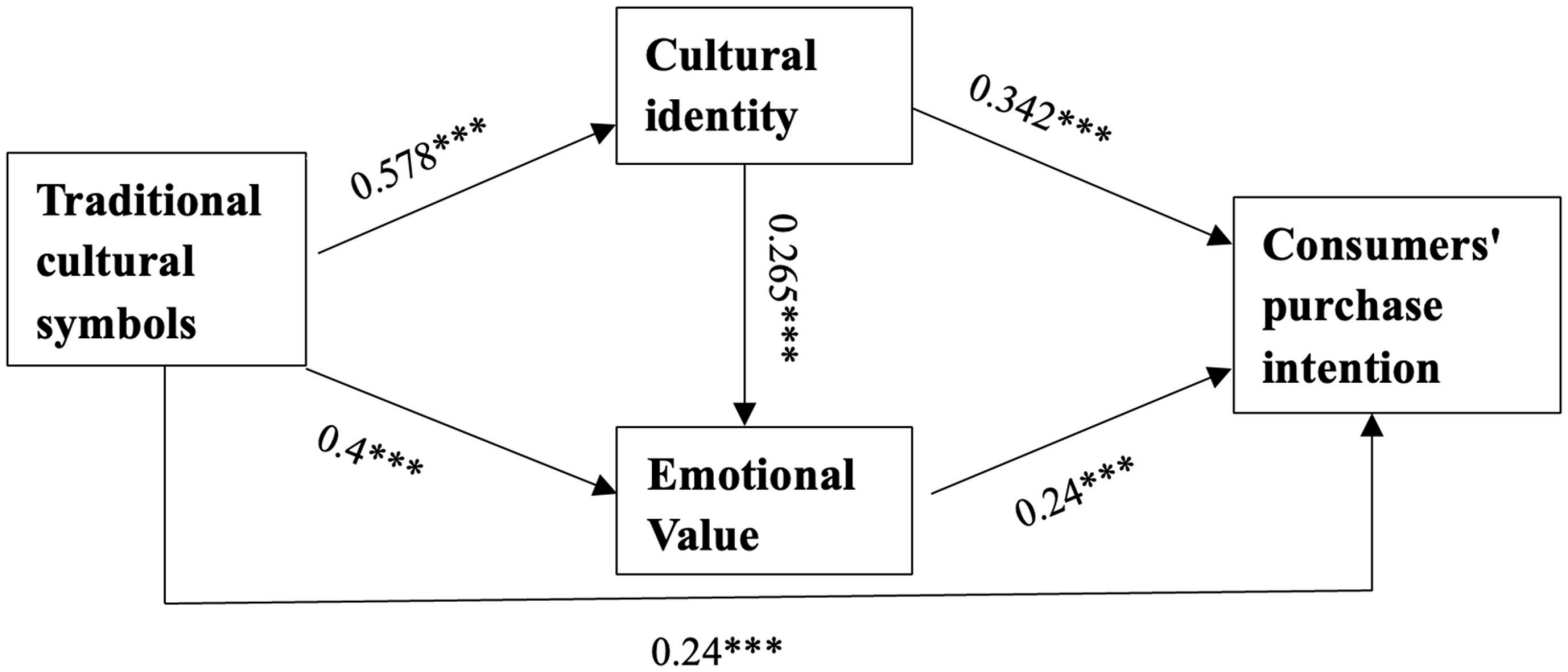
The mechanism of traditional culture on purchase responses.

In summary, a model of the mechanism of the influence of traditional culture on consumers’ purchase intention can be obtained, the details were shown in the Figure 3.

## Discussion

### Research conclusion

This study aims to explore the influence mechanism of traditional culture on the consumption willingness of the “National tide” from the perspective of traditional cultural identity. Specifically, we examined the association between two independent variables, traditional cultural symbols and cultural identity, and their association with emotional identity. Finally, we examine direct and indirect associations of emotional identity and cultural identity with consumers’ purchase intention. In the process, we combined cultural identity theory and the TPB model, and empirically tested seven hypotheses, drawing the following conclusions: First, the cognition of traditional cultural symbols and cultural identity has a direct and significant impact on the emotional value thereby, eliciting consumers’ purchase intention. Our findings support hypotheses H1 and H2, and H3 with *p* < 0.05. This shows that the perceived traditional cultural symbols have a significant positive impact on consumers’ traditional cultural identity and emotional values, and on the other hand, it also has a direct impact on the purchase intention, but it is not significant. The stronger consumers’ cognition of traditional cultural symbols, the stronger their cultural identity and emotional value. This result is consistent with previous research on symbols and identity. For example, Perach and Limbu (2022) report that masks with cultural symbols can increase positive interpersonal perceptions of people whose symbols represent meaningful social identities during the COVID-19 pandemic. Moreover, it is not only the contemporary cultural symbols that can elicit identification but also identification with traditional cultural symbols in our society (Monge et al., 2021).

Our findings endorsed that traditional cultural symbols are closely related to consumers, their way of thinking, and their willingness to buy. Moreover, the influence of cultural symbols on consumers’ purchase intention does not need to be based on cultural identity, which is very common in the purchase of tourist souvenirs. This means that when consumers buy souvenirs, they represent regional cultural characteristics, and their commemorative significance mainly emphasizes the symbolic or cultural meaning given by symbols (Trinh et al., 2014), rather than the identification of a certain regional culture. The authenticity and foreignness of travel commodities play an important role in consumers’ purchasing decisions (Li and Katsumata, 2020; Nusrat et al., 2021). Therefore, symbols can directly influence consumers’ purchase intention. At the same time, the research results show that emotional value is a subjective, high-level need. The stronger the cognition of traditional cultural symbols, the higher the emotional value, and the emotional value plays a partial mediating role between the cognition of traditional cultural symbols and consumers’ purchase intention.

Second, traditional cultural symbols are directly and indirectly (i.e., through emotional value or cultural identity) positively associated with consumers’ purchase intention, also cultural identity is directly and indirectly (i.e., through emotional value) associated with consumer purchase intention. Finally, emotional values and cultural identity mediate the indirect effect of traditional cultural symbols and cultural identity on purchase intention. Emotional value is the mediating variable between consumers’ traditional cultural identity and purchase intention. This conclusion is consistent with the findings of national patriotic sentiment and national community identity and the mediating variables of domestic commodity awareness (Zhou et al., 2010; Liu et al., 2020). Under the influence of deepening the cognition of traditional symbols, the degree of traditional cultural identity is strengthened, thereby enhancing its emotional value. When consumers feel more and more strongly about traditional culture, they will have a better impression of products containing traditional cultural symbols.

## Implications

### Theoretical implication

The study has three main theoretical contributions. First, our study introduces the influence mechanism of traditional culture on consumers’ purchase intention, and explores the influence of traditional culture on consumers’ purchase intention from a psychological perspective. Secondly, our research extends the existing limited scale of traditional cultural identity, which not only confirms the relationship between external traditional cultural symbols and people’s self-identity, but also enhances emotional value, which is conducive to the inheritance and popularization of traditional culture. Third, this study has provided a novel perspective on the development of the national fashion brands. Our research results show that by strengthening the rational transformation of traditional cultural symbols and enhancing the emotional value of consumers, we can stimulate consumers’ attitudes toward consumerism and promote the stable development of the “National Tide” market.

### Practical implication

This study also makes several important practical contributions. First, our study results have provided a reference index for market optimization to improve the consumers’ willingness to purchase “National Tide” products. Designers and market planners of the “National Tide” brand should consider the influence factors of consumers’ identification with traditional culture on their purchase intentions.

Secondly, since traditional cultural symbols can directly affect consumers’ willingness to buy, designers should reasonably transform traditional cultural symbols into product design. For example, as a visual touch point for consumers, product design is the most important form of expressing the uniqueness of a product. At the same time, it will give consumers the most intuitive first impression (Wu et al., 2022). It is necessary to avoid the problem of product homogeneity caused by the accumulation of traditional cultural symbols and modular patchwork. Organizations should innovate and rationally transform traditions and effectively combine traditional culture with fashion trends, such as Japanese tourist souvenirs and creative cultural products from the Forbidden City. This is the only way the “National Tide” brand can exert the continuous attraction of traditional culture to consumers.

Third, our findings provide enlightenment for “National Tide” brand marketers to explore the spiritual connotation of traditional cultural symbols and enhance consumers’ cognition of the cultural attributes of “National Tide” products. In the reasonable transformation of figurative traditional cultural symbols, our research suggests that the spiritual connotation of non-material traditional cultural symbols should be fully explored, and abstract ideology should be applied to product design concepts to establish a sense of belonging at the level of consumers ‘perception. Our research points out that the global reputation of Japanese products lies not only in their superior quality but also in the spirit of Japanese craftsmanship.

Finally, our findings provide insights that marketers should incorporate into their marketing campaigns. As mentioned in the previous section, simply emphasizing traditional cultural identity in the marketing process is not enough to trigger consumers’ purchase intention. The need to enhance their emotional value, i.e., cultural belonging and pride, can effectively influence consumers’ purchase intention. Additionally, marketers can use digital marketing to increase consumer engagement. The active role of social media in product sales should be fully leveraged. For example, product design inspiration and ideas should be actively displayed and promoted on social media (Khalid et al., 2021a,b; Saleem et al., 2021). The display of digital social media can better deepen consumers’ understanding of the cultural connotation of brands and products, and enhance consumers’ traditional cultural identity and emotional value.

### Limitations and future directions

This study has the following limitations. First, the current study only examined the problem from the perspective of traditional cultural symbols, identities, emotions, etc., and does not consider other factors that affect the purchase intention of national fashion brands, such as conservatism, cultural adaptation, etc. (Schwartz et al., 2017), cultural openness sex and other relevant pre-existing variables (Shimp and Sharma, 1987; Zhang et al., 2022). Therefore, the generalizability of the study is limited. Future studies can integrate more variables for more systematic studies. Second, more than 80% of the sample in this study was 30 years old or younger. Although it is consistent with the age distribution of the “National Tide” consumer groups of Baidu 2021 National Tide Pride Search Big Data, stated that 74% of the brand consumers in the entire industry are born in the post-90s and post-00s, there may be deviations in the research results. Therefore, more diverse samples are needed to strengthen the findings. Future researchers should expand their sample selection to increase the explanatory power and generalizability of our findings, prevent bias in their sample selection, and improve the overall credibility of the study. In addition, the emotion of traditional culture is a kind of consciousness. As a nonfigurative psychological concept, it should be measured in a more systematic and advanced way than traditional questionnaires. Therefore, a psychological approach to measuring this concept is needed in future research.

## Data availability statement

The original contributions presented in the study are included in the article/supplementary material, further inquiries can be directed to the corresponding author.

## Author contributions

ZZ, HG, and XL contributed to the conceptualization of the study and data collection. ZZ and HG also contributed to writing the first draft of the manuscript. HG and XL ran the analyses and wrote the results section. All authors contributed to the article and approved the submitted version.

## Funding

This research was funded by the National Social Science Foundation of China (grant number 22CH187).

## Conflict of interest

The authors declare that the research was conducted in the absence of any commercial or financial relationships that could be construed as a potential conflict of interest.

## Publisher’s note

All claims expressed in this article are solely those of the authors and do not necessarily represent those of their affiliated organizations, or those of the publisher, the editors and the reviewers. Any product that may be evaluated in this article, or claim that may be made by its manufacturer, is not guaranteed or endorsed by the publisher.
